# Circulating Levels of miR-574-5p Are Associated with Neurological Outcome after Cardiac Arrest in Women: A Target Temperature Management (TTM) Trial Substudy

**DOI:** 10.1155/2019/1802879

**Published:** 2019-06-02

**Authors:** Adeline Boileau, Antonio Salgado Somoza, Josef Dankiewicz, Pascal Stammet, Patrik Gilje, David Erlinge, Christian Hassager, Matthew P. Wise, Michael Kuiper, Hans Friberg, Niklas Nielsen, Yvan Devaux, TTM-Trial Investigators on behalf of Cardiolinc Network

**Affiliations:** ^1^Cardiovascular Research Unit, Luxembourg Institute of Health, Strassen L-1445, Luxembourg; ^2^Department of Cardiology, Clinical Sciences, Lund University and Skane University Hospital, Lund SE-221 85, Sweden; ^3^Medical and Health Department, National Fire and Rescue Corps, Luxembourg L-2557, Luxembourg; ^4^Department of Cardiology B, The Heart Centre, Rigshospitalet University Hospital, Copenhagen 2100, Denmark; ^5^Department of Intensive Care, University Hospital of Wales, Cardiff CF144XW, UK; ^6^Department of Intensive Care, Leeuwarden Medical Centrum, Leeuwarden 8934, Netherlands; ^7^Department of Anesthesia and Intensive Care, Clinical Sciences, Lund University and Skane University Hospital, Lund SE-221 85, Sweden; ^8^Department of Anesthesia and Intensive Care, Clinical Sciences, Lund University and Helsingborg Hospital, Lund SE-25187, Sweden; ^9^Cardiolinc Network, Luxembourg Institute of Health, Luxembourg

## Abstract

**Purpose:**

Postresuscitation neuroprognostication is guided by neurophysiological tests, biomarker measurement, and clinical examination. Recent investigations suggest that circulating microRNAs (miRNA) may help in outcome prediction after cardiac arrest. We assessed the ability of miR-574-5p to predict neurological outcome after cardiac arrest, in a sex-specific manner.

**Methods:**

In this substudy of the Target Temperature Management (TTM) Trial, we enrolled 590 cardiac arrest patients for which blood samples were available. Expression levels of miR-574-5p were measured by quantitative PCR in plasma samples collected 48 h after cardiac arrest. The endpoint of the study was poor neurological outcome at 6 months (cerebral performance category scores 3 to 5).

**Results:**

Eighty-one percent of patients were men, and 49% had a poor neurological outcome. Circulating levels of miR-574-5p at 48 h were higher in patients with a poor neurological outcome at 6 months (*p* < 0.001), both in women and in men. Circulating levels of miR-574-5p were univariate predictors of neurological outcome (odds ratio (OR) [95% confidence interval (CI)]: 1.5 [1.26-1.78]). After adjustment with clinical variables and NSE, circulating levels of miR-574-5p predicted neurological outcome in women (OR [95% CI]: 1.9 [1.09-3.45]), but not in men (OR [95% CI]: 1.0 [0.74-1.28]).

**Conclusion:**

miR-574-5p is associated with neurological outcome after cardiac arrest in women.

## 1. Introduction

Out-of-hospital cardiac arrest (OHCA) is a devastating condition, with overall survival rates lower than 10% [1]. Survival post-OHCA is associated with age, bystander cardiopulmonary resuscitation (CPR), type of first monitored rhythm, and time from cardiac arrest (CA) to the return of spontaneous circulation (ROSC) [2, 3]. Whether survival rate differs between men and women is not clear although several studies reported that women had higher survival rates than men [4].

It is well documented that the brain is highly sensitive to ischemia, and half of OHCA survivors suffer neurological damage which impacts their quality of life and survival [5]. Currently, most deaths after OHCA occur after withdrawal of life-supporting therapies in patients with severe and irreversible neurological sequelae [6]. The decision to withdraw life-supporting therapies is currently based on a multimodal approach including clinical examination, electrophysiological tests (absence of somatosensory evoked potential), electroencephalography, brain imaging, and assessment of protein biomarkers such as neuron-specific enolase (NSE) and S100b [7]. Cardiac biomarkers such as N-terminal probrain natriuretic peptide (NT-proBNP) and high-sensitive cardiac troponin T (hs-TnT) are associated with neurological outcome and death after OHCA but are not included in the guidelines [7–9]. Despite this multimodal approach, predicting outcome after OHCA, especially at an early stage and in patients with moderate brain damage, is challenging and would benefit from novel biomarkers.

MicroRNAs (miRNA) are small single-stranded RNA molecules that regulate gene expression and are involved in multiple pathophysiological processes. As they circulate in the blood and reflect disease status, they are considered promising biomarkers towards personalized medicine [10]. Several circulating miRNA have been shown to be associated with outcome after OHCA [11]. Previous studies showed associations between circulating levels of miR-21, miR-124-3p, and miR-122-5p and neurological outcome after OHCA [12–15]. The ability of brain-enriched miR-124-3p to predict outcome after OHCA has been validated in a substudy of the large TTM trial [16]. A combined use of miR-124-3p and miR-122-5p improved the outcome prediction in the same cohort [13].

We hereby aimed to extend previous investigations to novel miRNA which may provide an incremental predictive value. We focused on miR-574-5p,which is upregulated in the blood and heart tissue from patients with ischemic heart disease [17, 18] and in atrial tissues from patients with atrial fibrillation [19], two frequent causes of CA [17, 20, 21]. Of note, miR-574-5p is upregulated in the blood after intracerebral haemorrhage independently of the sex and, after ischemic stroke, specifically in men [22]. miR-574-5p is also upregulated by oestradiol treatment in breast cancer cells MCF-7 [20]. Hence, we centred our attention on sex differences, since the knowledge of the effect of sex on prediction modalities after OHCA is limited.

## 2. Materials and Methods

### 2.1. Patients

Nine hundred and thirty-nine unconscious adults admitted to an intensive care unit after an OHCA of presumed cardiac cause were enrolled in the TTM trial, in 36 recruiting centres from November 2010 to July 2013. The trial is aimed at evaluating the potential benefit of a targeted temperature management at 33°C compared to 36°C [23]. The TTM trial and collection of blood samples in participating countries was approved by ethical committees of each participating country and fulfils the declaration of Helsinki [24]. The trial is accessible at www.clinicaltrials.gov (NCT01020916), and the protocol of the trial is accessible at https://clinicaltrials.gov/ct2/show/NCT01020916?term=ttm-trial&rank=1. The design and protocol including statistical analysis, results, and interpretations of the results of the trial have been previously published [23, 25, 26]. Blood samples were collected at each site and centrally stored at the Integrated Biobank of Luxembourg, in compliance with the International Society for Biological and Environmental Repositories Best Practices and with International Organization for Standards (ISO 9001:2008, 17025:2005 and NF S96-900:2011).

### 2.2. Endpoints

In the present substudy, the endpoint was a poor neurological outcome at 6 months after OHCA as assessed with the cerebral performance category (CPC) scale [27]. CPC scores 1 and 2 are considered a good neurological outcome. CPC scores 3 to 5 are considered a poor neurological outcome. For each patient, the CPC score was measured as indicated in the TTM trial protocol [25].

### 2.3. Measurement of miRNA Levels

Samples recovered 48 h after ROSC were used to measure circulating levels of miRNA by quantitative PCR as previously described and as detailed in Supplementary Material [13, 16].

### 2.4. Measurement of NSE, hs-TnT, S100b, and NT-proBNP Levels

Six months after the end of the trial, a core laboratory measured NSE, hs-TnT, S100b, and NT-proBNP levels in serum samples recovered 48 h after OHCA, as previously described [8, 9, 28, 29].

### 2.5. Statistical Analysis

For demographic and clinical data, the Mann-Whitney test was used to compare two groups of continuous variables. The Chi-square test or the Fisher exact tests were used to compare two groups of categorical variables. A *p* value < 0.05 was considered statistically significant.

The Mann-Whitney test was used to compare miR-574-5p levels between two groups of patients. The Spearman correlation test on ranks was used to correlate miR-574-5p levels with age, levels of NSE, S100b, NT-proBNP, hs-TnT, miR-122-5p, and miR-124-3p.

For the prediction of neurological outcome, univariate and multivariable analysis with logistic regression allowed to estimate the association between miR-574-5p levels (log10-transformed and scaled) and neurological outcome at 6 months after OHCA, which was dichotomized: CPC 1 or 2 was considered a good outcome (0 value), and CPC 3, 4, or 5 was considered a poor outcome (1 value). 150-fold multiple imputation was used for missing values (51 values for NSE, 36 values for lactate). Odds ratio (OR) and 95% confidence intervals (CI) were computed for an increase of 1 unit for continuous variables and were centred and scaled. The Akaike information criterion (AIC) was used to estimate the prediction value of multivariable models: a low AIC indicates a better model fit. The likelihood ratio test was used to compare two AIC values. The AIC is penalized by the number of variables included in the model allowing to avoid model overfitting due to the multiplication of covariates. The incremental predictive value of miR-574-5p to the baseline model was evaluated by a decrease of AIC and the integrated discrimination improvement (IDI).

SigmaPlot version 12.5 was used for statistical analysis related to descriptive results such as the demographic and clinic feature of the patients, comparison between two groups of patients, correlations, and logistic regression. R software was used with the following packages (PredictABEL, lmtest) for univariate and multivariable analysis.

## 3. Results

### 3.1. Patient Selection and Characteristics

**Figure 1:**
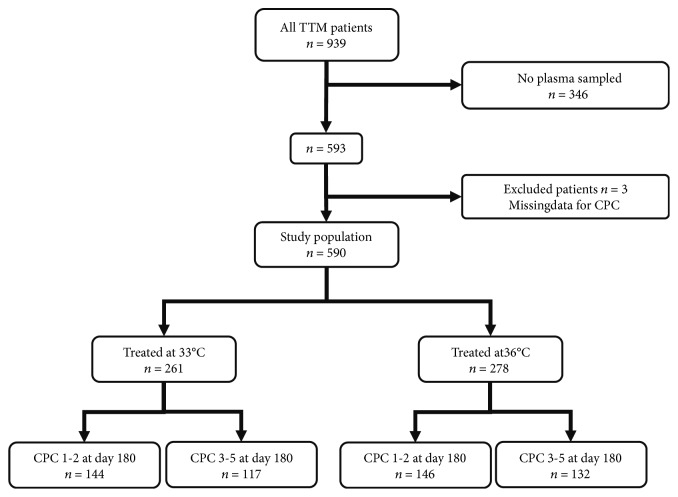
Study workflow.

A study design chart is available (Figure 1). Among the 939 patients of the TTM trial, plasma samples were available for 593 patients enrolled in 29 of the 36 recruiting centres. Three of these patients were excluded because of missing CPC data, allowing the inclusion of 590 patients in the present substudy. There was no difference in demographic and clinical features between the whole TTM cohort and the present substudy cohort (Supplementary Table 1), apart from a higher prevalence of alcohol abuse in the TTM cohort as compared to the present substudy (4% vs. 1.9%, respectively).

The demographic and clinical characteristics of the study population are presented with a comparison between patients with good neurological outcome (CPC 1-2) and patients with poor neurological outcome (CPC 3-5), for all patients (*n* = 590; Table 1) and separately for men (*n* = 481) and women (*n* = 109; Supplementary Table 2). Eighty-one percent of patients were men, and 49% had a poor neurological outcome. Patients with a poor neurological outcome were older; had more often comorbidities, longer time between CA and ROSC, and higher initial levels of serum lactate; and less frequently had bystander CPR compared to patients with a good neurological outcome. A higher proportion of patients with poor neurological outcome presented with shock at admission and had an initial nonshockable rhythm compared to patients with a good neurological outcome (Table 1). There was no difference between men and women (Supplementary Table 2).

### 3.2. Association between Circulating Levels of miR-574-5p and Patient Characteristics

We first sought to determine potential associations between circulating levels of miR-574-5p measured 48 h after OHCA and the age and sex of the patients. Levels of miR-574-5p were very moderately yet statistically significantly correlated with the age of all patients (*r* = 0.16, *p* < 0.001, Supplementary Figure 1a), in men (*r* = 0.16, *p* < 0.001, Supplementary Figure 1b) but not in women (*r* = 0.129, *p* = 0.192, Supplementary Figure 1c). There was no significant difference in miR-574-5p levels between men and women (Supplementary Figure 1d).

### 3.3. Circulating Levels of miR-574-5p according to Neurological Outcome and Temperature

**Figure 2:**
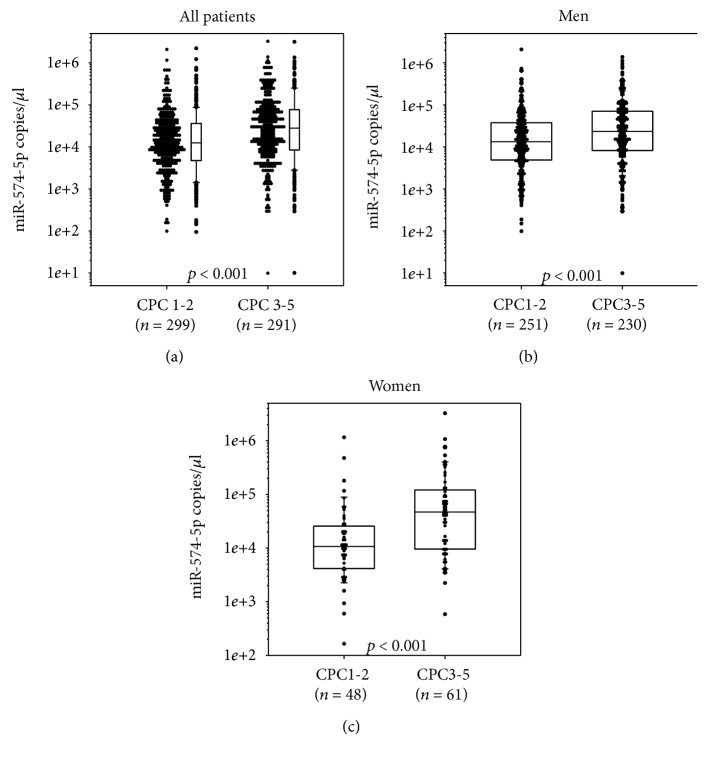
Plasma levels of miR-574-5p according to neurological outcome. Plasma levels of miR-574-5p were measured 48 h after the return of spontaneous circulation (ROSC) using quantitative PCR and were compared between patients with good (CPC 1-2) and poor (CPC 3-5) neurological outcomes. (a): 590 patients; (b): 481 men; (c): 109 women. Box plots represent the median and quartiles. Levels of miR-574-5p are expressed as the number of copies per microliter of plasma and are log-scaled. Plasma levels of miR-574-5p according to neurological outcome. Plasma levels of miR-574-5p were measured 48 h after the return of spontaneous circulation (ROSC) using quantitative PCR and were compared between patients with good (CPC 1-2) and poor (CPC 3-5) neurological outcomes. (a): 590 patients; (b): 481 men; (c): 109 women. Box plots represent the median and quartiles. Levels of miR-574-5p are expressed as the number of copies per microliter of plasma and are log-scaled.

Levels of miR-574-5p were higher in patients with poor neurological outcome (CPC 3-5, Figure 2(a)), independently of sex (Figures 2(b) and 2(c)) and of the targeted temperature management regimen (33°C vs. 36°C, Supplementary Figure 2a-f). Interestingly, levels of miR-574-5p were higher in patients treated at 33°C (Supplementary Figure 2g), in both women and men (Supplementary Figure 2h-i).

### 3.4. Sex-Specific Association between miR-574-5p Levels and Neurological Outcome

Levels of miR-574-5p measured 48 h after OHCA were univariate predictors of neurological outcome in all patients (OR [95% CI]: 1.50 [1.26-1.78], Supplementary Table.  3), in men (OR [95% CI]: 1.36 [1.13-1.64]; Supplementary Table.  3) and in women (OR [95% CI]: 2.28 [1.44-3.60]; Supplementary Table.  3). Consistent with past studies [13, 16, 28, 29], multivariable analyses included the following variables: age, sex (female), time from CA to ROSC, CPR, first monitored rhythm, circulatory shock on admission, initial serum lactate levels, NSE levels at 48 h, targeted temperature regimen, and miR-574-5p levels.

**Figure 3:**
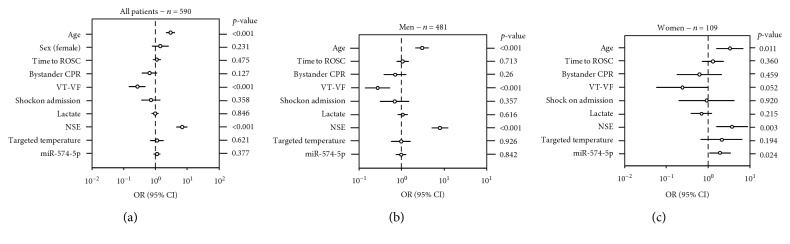
Sex-specific association between miR-574-5p levels and neurological outcome. Multivariable analyses (a–c) of the association between plasma miR-574-5p levels measured 48 h after OHCA and neurological outcome in all 590 patients (a), 481 men (b) and 109 women (c). Odds ratios (OR) ± 95% confidence intervals (95% CI) are shown for the prediction of poor neurological outcome (CPC 3-5) 6 months after OHCA. Variables included in the models: age, sex (female), time from cardiac arrest to return of spontaneous circulation (ROSC), bystander cardiopulmonary resuscitation (CPR), first monitored rhythm (ventricular tachycardia- (VT-) ventricular fibrillation (VF)), circulatory shock on admission, initial serum lactate levels, NSE levels at 48 h, targeted temperature regimen, and miR-574-5p levels.

As shown in Figure 3(a), age and NSE were significant predictors of neurological outcome. After adjustment with demographic and clinical variables, miR-574-5p remained an independent predictor of neurological outcome in women (OR [95% CI]: 1.9 [1.09-3.45], Figure 3(c)) but lost significance in men (OR [95% CI]: 1.0 [0.74-1.28], Figure 3(b)) and in all patients (OR [95% CI]: 1.1 [0.87-1.42], Figure 3(a)).

We next estimated the incremental predictive value of miR-574-5p to a baseline model including all variables included in multivariable analyses. The AIC and the IDI were calculated, bearing in mind that a lower AIC and a higher IDI indicate a better predictive value. Of note, we chose to calculate the AIC instead of the area under the curve to avoid model overfitting. Adding miR-574-5p to the baseline clinical model improved the prediction of neurological outcome in women, as attested by a significant decrease of AIC (*p* = 0.018) and an IDI of 0.04 [0.007-0.079] (Table 2). No incremental value was found in men or in all patients (Table 2).

### 3.5. Association between Circulating Levels of miR-574-5p and Markers of Neurological and Cardiac Damage

For all patients, and independently of sex, we observed a modest but significant correlation between miR-574-5p and NSE levels (*r* = 0.24, *p* < 0.001), as well as S100b levels (*r* = 0.29, *p* < 0.001, Supplementary Table 4). Circulating levels of miR-574-5p were modestly correlated with NT-proBNP levels (*r* = 0.17, *p* < 0.001) and hs-TnT levels (*r* = 0.20, *p* < 0.001; Supplementary Table 4). Interestingly, for all these markers of neurological and cardiac injury, correlations with miR-574-5p levels were slightly higher for women.

### 3.6. Association between Circulating Levels of miR-574-5p, miR-124-3p, and miR-122-5p

Levels of miR-574-5p were not correlated with circulating levels of miR-122-5p (*r* = 0.06, *p* = 0.19), but were positively correlated with miR-124-3p levels (*r* = 0.29, *p* < 0.001), independently of sex (Supplementary Table 4).

## 4. Discussion

This substudy of the TTM trial highlighted an association between circulating levels of miR-574-5p measured 48 h after OHCA and patient outcome. More specifically, we observed that this miRNA was an independent predictor of 6-month neurological outcome in women, but not in men.

We focused on miR-574-5p in the present study because it has been reported to be upregulated in plasma and cardiac samples from patients with ischemic heart disease, a frequent cause of CA [17, 18, 21]. However, circulating levels of miR-574-5p were only modestly correlated with the cardiac markers NT-proBNP and hs-TnT. This might be due to the inclusion of all OHCA of cardiac origin patients in the TTM trial, independently of the presence of ischemia.

Currently, miR-574-5p is not considered organ- or tissue-specific. It is expressed in the human heart and liver [30] and also in different cancer cell lines and adipose cells [31–33]. In mice, miR-574-5p plays different roles in the brain. It promotes the differentiation of neural progenitor cells into neurons [34], and levels of miR-574-5p were decreased in the brain of mice following injury by exposure to fine particles. Increased levels of miR-574-5p restored synaptic and cognitive impairment caused by fine particle exposure [35]. On the other hand, patients suffering of intracerebral haemorrhage showed an upregulation of circulating miR-574-5p levels, independently of sex [22]. In our study, circulating levels of miR-574-5p were weakly correlated with NSE and S100b levels. NSE is known to be expressed by neurons whereas S100b is expressed by glial cells. Both are released after brain injury following CA or in other neurological conditions such as traumatic brain injury [28, 29, 36, 37]. Our observations do not confirm that miR-574-5p only originates from the brain. The association between miR-574-5p and ischemia in different contexts (cerebral and cardiac) suggests that circulating levels of miR-574-5p may originate from different organs during or after ischemic-reperfusion injuries simultaneously after CA. Further animal experiments would be needed to test whether miR-574-5p is released from the brain and/or from other organs simultaneously after CA. Such studies would allow characterization of the tissue and cellular origin of miR-574-5p which remains poorly known.

Other miRNA have been previously studied in the context of OHCA, a number of them showing potential as prognostic indicators [11]. In the TTM cohort, both miR-124-3p and miR-122-5p showed strong associations with neurological outcome [13, 16]. miR-124-3p is enriched in the brain. The weak correlation between miR-574-5p and miR-124-3p do not support the possibility that miR-574-5p could be exclusively released from the injured brain after OHCA. Since miR-122-5p originates from the liver, the absence of correlation between miR-122-5p and miR-574-5p levels suggests that circulating levels of miR-574-5p are not only released by the liver, hence strengthening the assumption that miR-574-5p could be released simultaneously by several organs. In previous smaller-scale studies, miR-21 and miR-122-5p were also considered potential prognostic biomarkers [12, 14], although a recent small trial did not report significant associations between admission levels of miR-122-5p and all-cause mortality [15]. This lack of association might be due to the time of measurement (admission vs. 48 h post OHCA), and this highlights the need for future studies with serial assessment of miRNA, from admission to a few days after OHCA. This would determine the kinetics of miRNA release after OHCA as well as the optimal time point(s) for measurement, as studies on the kinetics of miRNA levels after OHCA are sparse.

In our study, circulating levels of miR-574-5p were higher in patients of the 33°C group. Hypothermia at 33°C induces lower clearance than hypothermia at 36°C [38], which could be involved in this upregulation of miR-574-5p. Simultaneously, Eskla et al. showed that, in HeLa cells, hypothermia at 32°C for 24h increased the expression of hypoxia-inducible factor- (HIF-) 1a [39], and another study showed that overexpression of HIF-1a led to miR-574-5p overexpression [40]. These results suggest HIF-1a may play a role in the higher levels of miR-574-5p observed in patients of the 33°C group. The exact pathway (and the clinical or neurological significance) leading to increased levels of miR-574-5p in the circulation after OHCA remains to be elucidated.

Sex disparities have been reported in the context of CA, for instance, a lower proportion of witnessed OHCA occurring in women or the fact that women have less OHCA from cardiac aetiology than men [41, 42]. Differences in treatment modalities, such as the utilization of coronary angiography, have been reported [43, 44]. Despite these, and after adjustment with confounders, no significant difference in mortality between men and women has been reported [44, 45], including in the TTM trial [41]. This is consistent with our present substudy in which sex was not associated with neurological outcome. We report here for the first time a sex-specific association between a candidate biomarker and outcome after OHCA. More specifically, we present data suggesting a prognostic value of miR-574-5p in women but not in men.

Patients after CA have higher blood levels of oestradiol than healthy controls [46] and miR-574-5p expression upregulated by oestradiol treatment *in vitro* [20]. This may suggest that higher levels of oestradiol in the blood may increase miR-574-5p expression, which would lead to higher circulating levels of miR-574-5p in women. Further studies are required to define a possible functional link between miR-574-5p and oestradiol which may explain the different prognostic value of miR-574-5p observed in the present study between men and women.

From a clinical point of view, predicting neurological outcome after OHCA represents a step forward towards personalized medicine. Predicting outcome at an early stage after OHCA would allow clinicians to optimize therapies in patients who would mostly likely benefit while guiding early decision making in patients with irreversible and severe neurological sequelae, thereby avoiding long and painful waiting periods for relatives [11]. Novel biomarkers will increase the accuracy of current multimodal prediction tools, and circulating miRNA may have the potential to attain an optimal predictive value. It will be important to conduct future studies in a sex-specific manner to avoid extrapolating results obtained on a mainly male population to women.

The limitations of this study are as follows: First, the choice of miR-574-5p relied on a known association with ischemic heart disease and atrial fibrillation while many other miRNA are regulated in the ischemic heart and may also deserve further investigation in the context of OHCA [10]. Second, we did not determine the cellular origin of miR-574-5p, which should be further investigated. Third, miR-574-5p was measured at a single time point. Fourth, our patient population contained only 109 women compared to 481 men, a difference which decreased the power of the study and emphasizes the need for additional validation.

## 5. Conclusion

We identified miR-574-5p as a female-specific predictor of neurological outcome after OHCA. Our data require further independent and large-scale testing of the ability of miR-574-5p to predict outcome after OHCA, focusing on sex disparities.

## Figures and Tables

**Table 1:** Demographic and clinical features of the 590 patients included in the present substudy.

	All patients (*n* = 590)	Good outcome (*n* = 299)	Poor outcome (*n* = 291)	*p* value (Good vs. poor)
Age (years)	65 (20-94)	61 (20-90)	68 (35-94)	**<0.001**
Comorbidities				
Hypertension	240 (41%)	102 (34%)	138 (47%)	**0.001**
Diabetes mellitus	86 (15%)	34 (11%)	52 (18%)	**0.034**
Known ischemic heart disease	163 (28%)	67 (22%)	96 (33%)	**0.005**
Previous MI	118 (20%)	48 (16%)	70 (24%)	**0.020**
Heart Failure	36 (6%)	9 (3%)	27 (9%)	**0.003**
COPD	55 (9%)	18 (6%)	37 (13%)	**0.008**
Renal failure	5 (1%)	1 (0%)	4 (1%)	0.353
Previous cerebral stroke	50 (8%)	19 (6%)	31 (11%)	0.084
Alcohol abuse	11 (2%)	4 (1%)	7 (2%)	0.513
First monitored rhythm				**<0.001**
VF or nonperfusing VT	467 (79%)	276 (92%)	191 (66%)	
Asystole or PEA	104 (18%)	16 (5%)	88 (30%)	
ROSC after bystander defibrillation	7 (1%)	5 (2%)	2 (1%)	
Unknown	12 (2%)	2 (1%)	10 (3%)	
Witnessed arrest	529 (90%)	276 (92%)	253 (87%)	**0.045**
Bystander CPR	433 (73%)	241 (81%)	192 (66%)	**<0.001**
Time from CA to ROSC (min)	25 (0-170)	20 (0-160)	30 (0-170)	**<0.001**
Initial serum lactate (mmol/l)	6.1 (0.5-25)	5.2 (0.5-20)	6.7 (0.5-25)	**<0.001**
Shock on admission	76 (13%)	27 (9%)	49 (17%)	**0.007**

Continuous variables are indicated as the median (range), and categorical variables are indicated as the number (frequency). CA: cardiac arrest; COPD: chronic obstructive pulmonary disease; CPR: cardiopulmonary resuscitation; MI: myocardial infarction; PEA: pulseless electric activity; ROSC: return of spontaneously circulation; VF: ventricular fibrillation; VT: ventricular tachycardia. Good outcome is CPC 1 or 2. Poor outcome is CPC 3, 4, or 5. Missing data: heart failure status for 2 patients, ischemic heart disease status for 1 patient, hypertension status for 1 patient, previous cerebral stroke status for 1 patient, diabetes mellitus status for 3 patients, alcohol abuse status for 1 patient, and lactate levels for 36 patients. *p* values < 0.05 were considered statistically significant and are in bold.

**Table 2:** Added value of miR-574-5p to predict neurological outcome in all patients and in men and women separately.

Models	AIC	*p* value	IDI [95% CI]	*p* value
All patients (*n* = 590)				
Baseline model	493.7			
Baseline model + miR-574-5p	494.9	0.376 (vs. baseline)	0.0009 [-0.0016; 0.0035]	0.465
Men (*n* = 481)				
Baseline model	395.2			
Baseline model + miR-574-5p	397.1	0.842 (vs. baseline)	0.0002 [-0.0005; 0.0009]	0.644
Women (*n* = 109)				
Baseline model	109.3			
Baseline model + miR-574-5p	105.7	**0.018** (vs. baseline)	0.0433 [0.0071; 0.0794]	**0.019**

The baseline model includes age, sex, bystander cardiopulmonary resuscitation (CPR), first monitored rhythm, time from cardiac arrest to ROSC, initial serum lactate levels, shock on admission, NSE levels at 48 h, and targeted temperature regimen. Log10-transformed miR-574-5 *p* values were used in these analyses. AIC: Akaike information criteria. A lower AIC indicates a better predictive value. IDI: integrated discrimination improvement. A higher IDI indicates a better predictive value. The statistical significance was assessed using the likelihood ratio test. A *p* value < 0.05 was considered significant and is highlighted in bold.

## Data Availability

The demographical, clinical, and microRNA expression data used to support the findings of this study are available from the corresponding author upon request. The results of univariate and multivariable analyses are included within the article and the supplementary information file.

## Abbreviations

AIC: Akaike information criteria
CA: Cardiac arrest
CI: Confidence interval
CPC: Cerebral performance category
HIF-1a: Hypoxia-inducible factor-1a
hs-TnT: High-sensitive cardiac troponin
IDI: Integrated discrimination improvement
miRNA: MicroRNA
NSE: Neuron-specific enolase
NT-proBNP: N-terminal probrain natriuretic peptide
OHCA: Out-of-hospital cardiac arrest
OR: Odds ratio
ROSC: Return of spontaneous circulation
TTM: Targeted temperature management.

## Supplementary Materials

Supplementary Materials Supplementary Table 1: comparison between demographic and clinical features of the patients included in the present substudy and the patients of the whole TTM cohort. Supplementary Table 2: demographic and clinical features of the 590 patients included in the present substudy separated by sex. Supplementary Table 3: univariate association between demographic and clinical characteristics, miR-574-5p levels, and neurological outcome at 6 months after CA in all 590 patients, 481 men and 109 women. Supplementary Table 4: correlation between levels of markers of neurological and cardiac damage, miR-122-5p, miR-124-3p, and miR-574-5p. Supplementary Figure 1: association between circulating levels of miR-574-5p and age and sex. Supplementary Figure 2: circulating levels of miR-574-5p according to targeted temperature management regimen and neurological outcome for all patients (a, d, g), men (b, e, h) and women (c, f, i).
