# Supplementary material for: Circulating Levels of miR-574-5p Are Associated with Neurological Outcome after Cardiac Arrest in Women: A Target Temperature Management (TTM) Trial Substudy
Source: Dis Markers. 2019 Jun 2;2019:1802879. doi: 10.1155/2019/1802879 (PMC6589199; doi:10.1155/2019/1802879)
Supplement: Supplementary Materials — Supplementary Table 1: comparison between demographic and clinical features of the patients included in the present substudy and the patients of the whole TTM cohort. Supplementary Table 2: demographic and clinical features of the 590 patients included in the present substudy separated by sex. Supplementary Table 3: univariate association between demographic and clinical characteristics, miR-574-5p levels, and neurological outcome at 6 months after CA in all 590 patients, 481 men and 109 women. Supplementary Table 4: correlation between levels of markers of neurological and cardiac damage, miR-122-5p, miR-124-3p, and miR-574-5p. Supplementary Figure 1: association between circulating levels of miR-574-5p and age and sex. Supplementary Figure 2: circulating levels of miR-574-5p according to targeted temperature management regimen and neurological outcome for all patients (a, d, g), men (b, e, h) and women (c, f, i). [file 1802879.f1.docx]

**Supplementary Materials**

**Methods**

1. **Measurement of circulating miR-574-5p levels**
   - *RNA extraction*

MiRNeasy Serum/plasma kit and the semi-automated QIAcube system (Qiagen, Hilden, Germany) were used to extract total RNA from 200µl of plasma, following the manufacturer’s instructions. Briefly, 200µl of plasma was mixed with 1ml of Qiazol. After 5 minutes of incubation, 3.5µl of spike-in working solution containing 1.6x10^8^ copies of cel-miR-39 per µl (Qiagen) was added to the sample, to correct for extraction efficiency. 200µl of chloroform were added and incubated for 3 minutes, followed by a centrifugation step at 10,000xg for 20min at 4°C. The aqueous phase obtained was recovered and was added on a silica column-based system, introduced into the QIAcube, which performed several washing steps with solutions containing increasing ethanol concentrations. 18µl of water were used to finally elute total RNA.

- - *RT-qPCR*

Reverse transcription was performed with miScript II reverse transcription kit (Qiagen) using 12µl of total RNA and HiSpec buffer. MiScript SYBR-green PCR kit (Qiagen), specific primers designed for miRNA (Qiagen) and CFX96 thermocycler (Bio-Rad) were used to perform quantitative PCR. Variations between PCR runs were adjusted using an inter-run calibrator in each PCR plate.

- - *Absolute quantification*

A stock solution (20µM, 1.204x10^13^ copies/µl) of synthetic miR-574-5p mimics (Qiagen) was used to prepare a working solution at 10^10^ copies/µl, followed by 10-fold serial dilution to obtain solutions ranging from 10^10^ copies/µl to 10^2^ copies/µl. 5µl of each solution of miR-574-5p mimic was reverse transcribed using the miScript II reverse transcription kit and HiSpec buffer (Qiagen). 50ng of carrier RNA (Qiagen) was used as negative reverse transcription control. Obtained cDNA was diluted 10-fold, and 4µl of diluted cDNA were used in quantitative PCR, performed with MiScript SYBR-green PCR kit (Qiagen). The CFX96 Manager 3.1 software (Bio-Rad) allowed the determination of the slope of the standard curve (b=-3.204) as well as the Y-axis intercept (m=42.996). MiR-574-5p quantity per well in PCR plate was determined using the following formula: 10^((Cq miR-574-5p-b)/m)^. MiR-574-5p quantity per samples was determined by multiplying this last value with a correction factor of 75 to account for dilutions during the process. This corrected value was divided by the starting volume of plasma (200µl) to obtain the number of copies of miR-574-5p per µl of plasma.

- - *Normalization*

Normalization of the number of copies of miR-574-5p by µl of plasma was performed by multiplication with a normalization factor, as described previously ^1^. This factor was obtained for each plasma sample using the formula: 1/[2^(median cel-miR-39 Cq value)-(cel-miR-39 Cq value of the given sample)^], in which the median cel-miR-39 Cq value was calculated from the median of cel-miR-39 Cq values of all samples to be compared.

Overall, the following formula was used to obtain normalized miR-574-5p values in copies of miR-574-5p per µl of plasma:

10^((Cq miR-574-5p-b)/m)^ x dilution factor x normalization factor;

in which dilution factor is 75/200

and normalization factor is 1/[2^(median cel-miR-39 Cq value)-(cel-miR-39 Cq value of the given sample)^].

**Supplementary Tables**

**Supplementary Table 1: Comparison between demographic and clinical features of the patients included in the present sub-study and the patients of the whole TTM cohort.**

| **Characteristic** | **TTM**  (n= 939) | **Sub-study**  (n= 590) | **P value** |
| --- | --- | --- | --- |
| **Age, years** | 65 (20-94) | 65 (20-94) | 0.939 |
| **Sex, female** | 178 (19%) | 109 (18.5%) | 0.867 |
| **Neurological outcome** |  |  |  |
| Good outcome (CPC 1-2) | 446 (47.5%) | 299 (50.8%) | 0.247 |
| **Co-morbidities** |  |  |  |
| Hypertension | 374 (40%) | 240 (40.8%) | 0.783 |
| Diabetes mellitus | 141 (15%) | 86 (14.6%) | 0.872 |
| Known ischemic heart disease | 260 (27.7%) | 163 (27.6%) | 0.974 |
| Previous MI | 193 (21%) | 118 (20%) | 0.844 |
| Heart Failure | 61 (6%) | 36 (6.1%) | 0.841 |
| COPD | 97 (10%) | 55 (9.3%) | 0.580 |
| Renal failure | 6 (1%) | 5 (0.8%) | 0.874 |
| Previous cerebral stroke | 73 (8%) | 50 (8.5%) | 0.694 |
| Alcohol abuse | 37 (4%) | 11 (1.9%) | **0.034** |
| **First monitored rhythm** |  |  | 0.905 |
| VF or non-perfusing VT | 729 (78%) | 467 (79.4%) |  |
| Asystole or PEA | 178 (19%) | 104 (17.6%) |  |
| ROSC after bystander defibrillation | 13 (1%) | 7 (1.2%) |  |
| Unknown | 19 (2%) | 12 (2%) |  |
| **Witnessed arrest** | 838 (89%) | 529 (90%) | 0.863 |
| **Bystander CPR** | 683 (73%) | 433 (73.4%) | 0.825 |
| **Time from CA to ROSC, min** | 25 (0-170) | 25 (0-170) | 0.324 |
| **Initial serum lactate (mmol/l)** | 6.0 (0.5-25) | 6.1 (0.5-25) | 0.609 |
| **Shock on admission** | 137 (15%) | 76 (12.9%) | 0.388 |
|  |  |  |  |

Continuous variables are indicated as median (range) and categorical variables are indicated as number (frequency). CA: cardiac arrest; COPD: chronic obstructive pulmonary disease; CPR: cardio-pulmonary resuscitation; MI: myocardial infarction; PEA: pulseless electric activity; ROSC: return of spontaneously circulation; VF: ventricular fibrillation; VT: ventricular tachycardia. Missing information: CPC status for 6 patients (in the TTM cohort, none in the sub-study), hypertension status for 4 patients, diabetes mellitus status for 7 patients, ischemic heart disease status for 3 patients, previous MI status for 2 patients, heart failure status for 3 patients, COPD status for 1 patient, renal status for 1 patient, previous cerebral stroke status for 4 patients, alcohol abuse status for 2 patients, witnessed arrest status for 1 patient, bystander CPR status for 2 patients, time from CA to ROSC for 1 patient, initial serum lactate for 62 patients and shock on admission status for 1 patient.

**Supplementary Table 2.** **Demographic and clinical features of the 590 patients included in the present sub-study separated by sex**

|  | **MEN (n=481)** | | | | **WOMEN (n=109)** | | | | **P value (all men vs all women)** |
| --- | --- | --- | --- | --- | --- | --- | --- | --- | --- |
|  | **All men** | **Good outcome**  (n=251) | **Poor outcome**  (n= 230) | **P value**  (Good vs poor) | **All women** | **Good outcome**  (n=48) | **Poor outcome**  (n= 61) | **P value**  (Good vs poor) |  |
| **Age, years** | 65 (20-94) | 61 (20-90) | 68 (35-94) | **<0.001** | 66 (24-88) | 59 (28-88) | 69 (46-88) | **<0.001** | 0.410 |
| **Co-morbidities** |  |  |  |  |  |  |  |  |  |
| Hypertension | 193 (40%) | 86 (34%) | 107 (46%) | **0.007** | 47 (43%) | 16 (33%) | 31 (51%) | 0.102 | 0.641 |
| Diabetes mellitus | 68 (14%) | 29 (12%) | 39 (17%) | 0.108 | 18 (16%) | 5 (10%) | 13 (21%) | 0.194 | 0.628 |
| Known ischaemic heart disease | 139 (29%) | 60 (24%) | 79 (34%) | **0.014** | 24 (22%) | 7 (15%) | 17 (28%) | 0.153 | 0.183 |
| Previous MI | 102 (21%) | 44 (17%) | 58 (25%) | 0.051 | 16 (15%) | 4 (8%) | 12 (20%) | 0.165 | 0.160 |
| Heart Failure | 29 (6%) | 8 (3%) | 21 (9%) | **0.010** | 7 (6%) | 1 (2%) | 6 (10%) | 0.213 | 0.947 |
| COPD | 42 (9%) | 15 (6%) | 27 (12%) | **0.038** | 13 (12%) | 3 (6%) | 10 (16%) | 0.185 | 0.393 |
| Renal failure | 3 (1%) | 1 (0%) | 2 (1%) | 0.939 | 2 (2%) | 0 (0%) | 2 (3%) | 0.584 | 0.505 |
| Previous cerebral stroke | 38 (8%) | 15 (6%) | 23 (10%) | 0.139 | 12 (11%) | 4 (8%) | 8 (13%) | 0.629 | 0.389 |
| Alcohol abuse | 9 (2%) | 4 (2%) | 5 (2%) | 0.889 | 2 (2%) | 0 (0%) | 2 (3%) | 0.584 | 0.714 |
| **First monitored rhythm** |  |  |  | **<0.001** |  |  |  | **0.002** | 0.088 |
| VF or non-perfusing VT | 388 (81%) | 233 (93%) | 155 (67%) |  | 79 (72%) | 43 (90%) | 36 (59%) |  |  |
| Asystole or PEA | 77 (16%) | 12 (5%) | 65 (28%) |  | 27 (25%) | 4 (8%) | 23 (38%) |  |  |
| ROSC after bystander defibrillation | 7 (1%) | 5 (2%) | 2 (1%) |  | 0 (0%) | 0 (0%) | 0 (0%) |  |  |
| Unknown | 9 (2%) | 1 (0%) | 8 (3%) |  | 3 (3%) | 1 (2%) | 2 (3%) |  |  |
| **Witnessed arrest** | 437 (91%) | 233 (93%) | 204 (89%) | 0.158 | 92 (84%) | 43 (90%) | 49 (80%) | 0.291 | 0.068 |
| **Bystander CPR** | 358 (74%) | 204 (81%) | 154 (67%) | **<0.001** | 75 (69%) | 37 (77%) | 38 (62%) | 0.148 | 0.281 |
| **Time from CA to ROSC, min** | 25 (0-170) | 20 (0-160) | 30 (0-170) | **<0.001** | 23 (0-156) | 21 (0-90) | 25 (5-156) | **0.014** | 0.325 |
| **Initial serum lactate (mmol/l)** | 6.2 (0.5-25) | 5.3 (0.5-20) | 6.5 (0.5-25) | **0.002** | 6 (0.8-21) | 4.2 (1.2-19) | 6.9 (0.8-21) | 0.117 | 0.901 |
| **Shock on admission** | 60 (12%) | 23 (9%) | 37 (16%) | **0.031** | 16 (15%) | 4 (8%) | 12 (20%) | 0.165 | 0.644 |

Continuous variables are indicated as median (range) and categorical variables are indicated as number (frequency). CA: cardiac arrest; COPD: chronic obstructive pulmonary disease; CPR: cardio-pulmonary resuscitation; MI: myocardial infarction; PEA: pulseless electric activity; ROSC: return of spontaneously circulation; VF: ventricular fibrillation; VT: ventricular tachycardia. Good outcome is CPC 1 or 2, Poor outcome is CPC 3, 4 or 5. Missing data: heart failure status for 2 patients, ischaemic heart disease status for 1 patient, hypertension status for 1 patient, previous cerebral stroke status for 1 patient, diabetes mellitus status for 3 patients, alcohol abuse status for 1 patient and lactate levels for 36 patients. P values <0.05 were considered statistically significant and are in bold.

**Supplementary Table 3: Univariate association between demographic, clinical characteristic, miR-574-5p levels and neurological outcome at 6 months after CA in all 590 patients, 481 men and 109 women.** Odds ratios (OR) ± 95% confidence intervals (95% CI) are shown for the prediction of poor neurological outcome (CPC 3-5) 6 months after OHCA.

| **OR (95%CI)** | **All patients** | **Men** | **Women** |
| --- | --- | --- | --- |
| *Age* | 2.39 (1.91-2.98) | 2.27 (1.79-2.91) | 2.87 (1.69-4.87) |
| *Sex (Female)* | 1.39 (0.91-2.11) |  |  |
| *Time to ROSC* | 1.77 (1.47-2.13) | 1.81 (1.47-2.23) | 1.70 (1.13-2.56) |
| *Bystander CPR* | 0.47 (0.32-0.68) | 0.47 (0.31-0.71) | 0.49 (0.21-1.15) |
| *VT-VF* | 0.18 (0.11-0.28) | 0.19 (0.11-0.31) | 0.15 (0.05-0.42) |
| *Shock on admission* | 2.04 (1.24-3.37) | 1.90 (1.09-3.31) | 2.69 (0.81-8.97) |
| *Lactate* | 1.35 (1.14-1.59) | 1.38 (1.14-1.66) | 1.26 (0.87-1.82) |
| *NSE* | 5.77 (4.27-7.80) | 6.46 (4.55-9.16) | 3.95 (2.14-7.29) |
| *Targeted temperature* | 1.02 (0.73-1.40) | 0.96 (0.67-1.37) | 1.18 (0.54-2.56) |
| *miR-574-5p* | 1.50 (1.26-1.78) | 1.36 (1.13-1.64) | 2.28 (1.44-3.60) |

CPR: cardio-pulmonary resuscitation; NSE: neuron specific enolase; ROSC: return of spontaneously circulation; VF: ventricular fibrillation; VT: ventricular tachycardia.

**Supplementary Table 4: Correlation between levels of markers of neurological and cardiac damage, miR-122-5p, miR-124-3p and miR-574-5p.**

|  | **All patients** | **Men** | **Women** |
| --- | --- | --- | --- |
| *NSE* |  |  |  |
| r | **0.242** | **0.221** | **0.344** |
| p | **<0.001** | **<0.001** | **<0.001** |
| *S100b* |  |  |  |
| r | **0.287** | **0.245** | **0.457** |
| p | **<0.001** | **<0.001** | **<0.001** |
| *NT-proBNP* |  |  |  |
| r | **0.167** | **0.151** | **0.230** |
| p | **<0.001** | **0.001** | **0.02** |
| *hs-TnT* |  |  |  |
| r | **0.198** | **0.210** | **0.232** |
| p | **<0.001** | **<0.001** | **0.02** |
| *miR-122-5p* |  |  |  |
| r | 0.059 | 0.082 | -0.02 |
| p | 0.191 | 0.10 | 0.847 |
| *miR-124-3p* |  |  |  |
| r | **0.294** | **0.288** | **0.332** |
| p | **<0.001** | **<0.001** | **0.001** |

BNP: brain natriuretic peptide, hs-TnT: high sensitivity troponine T, NSE: neuron-specific enolase, r: correlation coefficient. Note that NSE values were missing for 51 patients (40 men and 11 women), S100b values were missing for 50 patients (40 men and 10 women) and that NT-proBNP and hs-TnT values were missing for 52 patients (41 men and 11 women). Note that miR-122-5p and miR-124-3p values were missing for 92 patients (78 men and 14 women).

**Supplementary Figure 1: Association between circulating levels of miR-574-5p and age and sex.** Spearman correlation between miR-574-5p levels and age for all patients (**a**), men (**b**) and women (**c**). **d.** Plasma levels of miR-574-5p according to sex. r: correlation coefficient

**Supplementary Figure 2: Circulating levels of miR-574-5p according to targeted temperature management regimen and neurological outcome for all patients (a, d, g), men (b, e, h) and women (c, f, i).** Plasma levels of miR-574-5p were measured 48h after ROSC using quantitative PCR in 590 patients (481 men and 109 women). Displayed are levels of miR-574-5p between patients with good and poor neurological outcome in the 33°C group (**a-c**) and in the 36°C group (**d-f**). **g-i.** Levels of miR-574-5p between patients treated at 33°C and patients treated at 36°C. Levels of miR-574-5p are expressed as number of copies per microliter of plasma and are log-scaled.

**References**

1. Mitchell, P.S., Parkin, R.K., Kroh, E.M., Fritz, B.R., Wyman, S.K., Pogosova-Agadjanyan, E.L., Peterson, A., Noteboom, J., O'Briant, K.C., Allen, A., Lin, D.W., Urban, N., Drescher, C.W., Knudsen, B.S., Stirewalt, D.L., Gentleman, R., Vessella, R.L., Nelson, P.S., Martin, D.B. and Tewari, M. (2008). Circulating microRNAs as stable blood-based markers for cancer detection. Proceedings of the National Academy of Sciences of the United States of America 105, 10513-10518.

2. Stammet, P., Collignon, O., Hassager, C., Wise, M.P., Hovdenes, J., Aneman, A., Horn, J., Devaux, Y., Erlinge, D., Kjaergaard, J., Gasche, Y., Wanscher, M., Cronberg, T., Friberg, H., Wetterslev, J., Pellis, T., Kuiper, M., Gilson, G., Nielsen, N. and Investigators, T.T.-T. (2015). Neuron-Specific Enolase as a Predictor of Death or Poor Neurological Outcome After Out-of-Hospital Cardiac Arrest and Targeted Temperature Management at 33 degrees C and 36 degrees C. Journal of the American College of Cardiology 65, 2104-2114.

3. Gilje, P., Koul, S., Thomsen, J.H., Devaux, Y., Friberg, H., Kuiper, M., Horn, J., Nielsen, N., Pellis, T., Stammet, P., Wise, M.P., Kjaergaard, J., Hassager, C., Erlinge, D. and group, T.T.M.s. (2016). High-sensitivity troponin-T as a prognostic marker after out-of-hospital cardiac arrest - A targeted temperature management (TTM) trial substudy. Resuscitation 107, 156-161.

4. Stammet, P., Dankiewicz, J., Nielsen, N., Fays, F., Collignon, O., Hassager, C., Wanscher, M., Unden, J., Wetterslev, J., Pellis, T., Aneman, A., Hovdenes, J., Wise, M.P., Gilson, G., Erlinge, D., Horn, J., Cronberg, T., Kuiper, M., Kjaergaard, J., Gasche, Y., Devaux, Y., Friberg, H. and Target Temperature Management after Out-of-Hospital Cardiac Arrest trial, i. (2017). Protein S100 as outcome predictor after out-of-hospital cardiac arrest and targeted temperature management at 33 degrees C and 36 degrees C. Crit Care 21, 153.

5. Frydland, M., Kjaergaard, J., Erlinge, D., Stammet, P., Nielsen, N., Wanscher, M., Pellis, T., Friberg, H., Hovdenes, J., Horn, J., Wetterslev, J., Thomsen, J.H., Bro-Jeppesen, J., Winther-Jensen, M., Wise, M.P., Kuiper, M., Cronberg, T., Gasche, Y., Devaux, Y., Aneman, A. and Hassager, C. (2016). Usefulness of Serum B-Type Natriuretic Peptide Levels in Comatose Patients Resuscitated from Out-of-Hospital Cardiac Arrest to Predict Outcome. The American journal of cardiology 118, 998-1005.
